# Estimating the beginning of the waterpipe epidemic in Syria

**DOI:** 10.1186/1471-2458-4-32

**Published:** 2004-08-04

**Authors:** Samer Rastam, Kenneth D Ward, Thomas Eissenberg, Wasim Maziak

**Affiliations:** 1 Syrian Center for Tobacco Studies, Aleppo, Syria; 2 Center for Community Health, University of Memphis, Memphis, USA; 3 Department of Psychology, Virginia Commonwealth University, Richmond, USA; 4 Institute of Epidemiology and Social Medicine, University of Muenster, Muenster, Germany

## Abstract

**Background:**

Waterpipe smoking is becoming a global public health problem, especially in the Eastern Mediterranean region (EMR).

**Methods:**

We try in this study, which is a cross sectional survey among a representative sample of waterpipe smokers in cafes/restaurants in Aleppo-Syria, to assess the time period for the beginning of this new smoking hype. We recruited 268 waterpipe smokers (161 men, 107 women; mean age ± standard deviation (SD) 30.1 ± 10.2, response rate 95.3%). Participants were divided into 4 birth cohorts (≤ 1960, 1961–1970, 1971–1980, >1980) and year of initiation of waterpipe smoking and daily cigarette smoking were plotted according to these birth cohorts.

**Results:**

Data indicate that unlike initiation of cigarette smoking, which shows a clear age-related pattern, the nineties was the starting point for most of waterpipe smoking implicating this time period for the beginning of the waterpipe epidemic in Syria.

**Conclusion:**

The introduction of new flavored and aromatic waterpipe tobacco (*Maassel*), and the proliferation of satellite and electronic media during the nineties may have helped spread the new hype all over the Arab World.

## Background

Waterpipe smoking is becoming increasingly a worldwide phenomenon, with populations in the Eastern Mediterranean region (EMR) being especially affected [1]. This centuries-old tobacco use method comes under many different names (e.g., shisha, hookah, narghile, arghile), shapes, and sizes, depending on the region, with the term waterpipe implying a unifying feature of all these forms; *the passage of smoke through water before inhalation by the smoker *[2], Recent evidence shows that a quarter of some populations in the EMR currently smoke the waterpipe [3]. This trend is worrisome because of tobacco's known harmful effects to human health, and because prevailing norms the EMR may put certain slices of the society at increased risk of acquiring the habit, particularly women and children [6,7]. Although research on the health effects of waterpipe is still scarce, preliminary evidence links waterpipe use to respiratory, cardiovascular, and cancer diseases [8-11].

Developing effective intervention strategies to curb this emerging public health problem requires clear understanding of factors influencing the take up of this habit, as well its time course [1]. According to waterpipe smokers, the recent resurgence in waterpipe popularity is due to the introduction of *Maassel *(a specially prepared tobacco with sweetened fruit flavors and mild aromatic smoke), the media, and social trends [6]. Understanding the context in which these factors operate as well as being able to follow the secular course of the waterpipe epidemic requires estimation of the time frame for the beginning of the waterpipe hype. In this study we try to identify this time frame as well as provide evidence for the increase in waterpipe smoking.

## Methods

The current analysis is drawn from a survey conducted in 2003 among a representative sample of waterpipe smokers visiting cafes/restaurants in Aleppo, Syria. The survey details can be found elsewhere [12], but briefly a cross sectional interviewer-administered survey was conducted in 17 randomly selected (out of total 112) café/restaurants in Aleppo, Syria. Overall, 268 waterpipe smokers were recruited (161 men and 107 women; mean age ± SD 30.1 ± 10.2, age range 18–68 years; response rate 95.3%). Participants were asked about their waterpipe use frequency, cigarette smoking status, current age, age of initiation of waterpipe smoking, and age of initiation of daily cigarette smoking.

The protocols and informed consent documents for this study were approved by the SCTS' IRB and the University of Memphis' IRB. The questionnaire was anonymous and informed consent was obtained prior to all interviews.

## Analysis

First, year of birth was calculated by subtracting current age of participant from the year of survey (2003), while year of initiation of waterpipe smoking and daily cigarette smoking were calculated by adding the age of initiation of smoking to the year of birth. Year of birth was then divided into four decade-long categories (people born in/before 1960; during 1961–1970; during 1971–1980; in/after 1981), and year of smoking initiation into three decade-long categories (initiation in/before 1990, initiation during 1991–2000, and initiation in/after 2001).

The Chi-Square test was used to assess differences between the three smoking initiation time-groups for each birth cohort, with *p *level <0.05 considered significant.

## Results and conclusions

**Figure 1:**
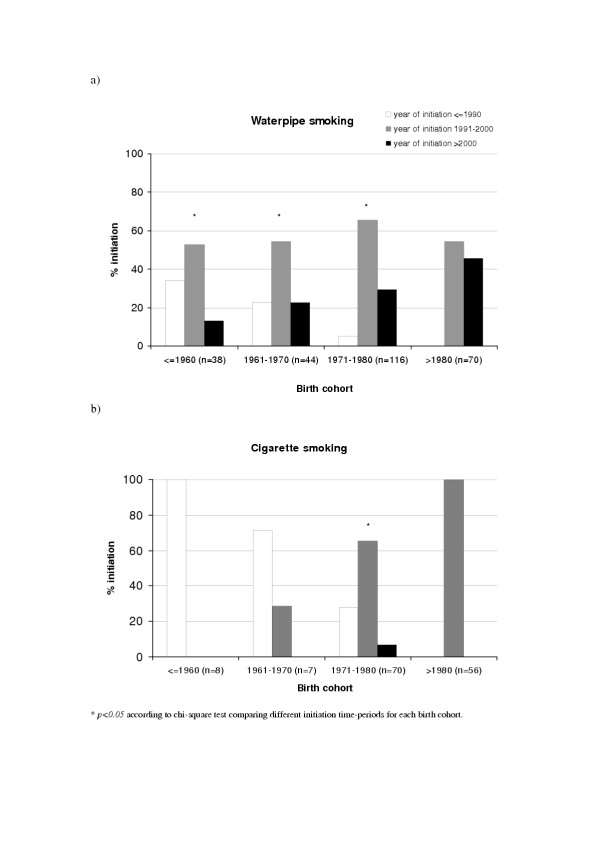
a: Shows the proportion of current waterpipe smokers of different birth cohorts according to their year of initiation categorized into three decade-long categories. b. The same parameters are shown for cigarette smoking.

**Figure 2:**
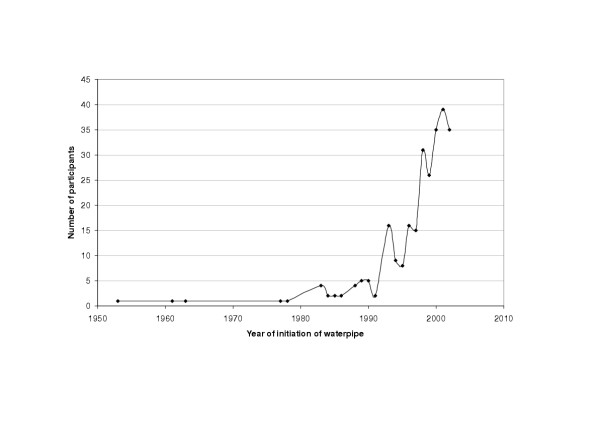
Shows number of study participants according to their year of initiation of waterpipe smoking.

Figure 1a,1b compares between year of initiation of waterpipe and cigarettes among study participants, respectively, according to their birth cohort. It shows that while cigarette initiation displays an age-related pattern with peak initiation of participants occurring during in their twenties, most of waterpipe initiation and for all birth cohorts is commencing in the 1990s. Figure 2, depicts number of waterpipe smokers according to their year of initiation, and suggests indirectly the rapid increasing trend of this smoking method. Taking into consideration possible limitations of this study, particularly the use of cross-sectional design to examine time trends and the relative youngness of the study sample, these results suggest that the current hype of waterpipe smoking is a recent one, commencing mostly in the nineties of the 20^th ^century, and showing an increasing trend. Based on these results and our previous data regarding factors related to the spread of waterpipe [6], we present the following scenario for the current surge of popularity of waterpipe smoking. In the nineties, *Maassel *was introduced [13] simplifying waterpipe preparation and attracting more people to its mild aromatic smoke. Out of curiosity about this new tobacco or of modeling of others, people started experimenting with the waterpipe. The increasing number of waterpipe users together with the spread of satellite channels and electronic media, occurring during the same time period, may have contributed further to the creation and spread of this new smoking trend. Since waterpipe smoke contains considerable amounts of the addictive substance nicotine [14,15], nicotine dependence can sustain the habit among experimenters creating a stable base of waterpipe smokers within the society and contributing further to its spread. It remains to be seen, the possible role of resurgence of local identities in contrast to western culture in the adoption of this "oriental" method of smoking. The dramatic increase of this addictive smoking method within a short period of time and its potential health risks mandate that active surveillance and in depth research into its risk profile should become a priority for health systems in the EMR. Policy makers should also be alerted to this eminent public health threat, which is so far escaping current regulations and restrictions imposed on cigarettes, such as the ban on advertisement and the inclusion of health warnings on waterpipe tobacco products.

## Competing interests

None declared.

## Authors' contributions

SR designed the study, conducted the analysis and wrote the first draft of the manuscript. KW and TE contributed to the design of survey and revision of the manuscript. WM contributed to the design of survey and wrote the final draft of the manuscript. All authors read and approved the final manuscript.

## Pre-publication history

The pre-publication history for this paper can be accessed here:


